# Effects of intensive rosuvastatin on ventricular remodeling and cardiac function in elderly patients with STEMI undergoing PCI

**DOI:** 10.3389/fcvm.2025.1638967

**Published:** 2025-08-22

**Authors:** Yiran Qin, Siyi Jin, Xusen Sun, Rong Luo, Haibo Liu

**Affiliations:** ^1^ Department of Cardiology, Qingpu Branch of Zhongshan Hospital Affiliated to Fudan University, Shanghai, China; ^2^ Department of Hospital Infection Management, Qingpu Branch of Zhongshan Hospital Affiliated to Fudan University, Shanghai, China

**Keywords:** rosuvastatin, ST-segment elevation myocardial infarction, inflammatory cytokines, ventricular remodeling, cardiac function

## Abstract

**Background:**

Acute myocardial infarction in the elderly often leads to significant left ventricular structural remodeling, which adversely affects prognosis. This study aims to evaluate the effects of intensive rosuvastatin therapy on markers of ventricular remodeling and cardiac function following percutaneous coronary intervention (PCI) in elderly patients with ST-segment elevation myocardial infarction (STEMI).

**Methods:**

This study enrolled 100 patients aged ≥60 years with STEMI who underwent emergency PCI. The patients were randomly assigned to either an intensive therapy group (*n* = 50), receiving rosuvastatin 20 mg/day, or a control group (*n* = 50), receiving 10 mg/day. Differences in lipid profiles, serum inflammatory markers, fibrosis indicators, and echocardiographic parameters were compared between the two groups before treatment and after 8 weeks of therapy.

**Results:**

After 8 weeks of treatment, the intensive group showed significantly reduced serum inflammatory levels compared to the control group, including C-reactive protein (CRP), interleukin-6 (IL-6), tumor necrosis factor-α (TNF-α), and intercellular adhesion molecule-1 (ICAM-1) (*P* < 0.05). Markers of ventricular remodeling also improved in the intensive group, with lower levels of N-terminal pro-B-type natriuretic peptide (NT-proBNP), galectin-3, and matrix metalloproteinase-9 (MMP-9) compared to the control group (*P* < 0.05), while levels of tissue inhibitor of metalloproteinases-4 (TIMP-4) were significantly higher (*P* < 0.05). Additionally, after treatment, the intensive group demonstrated significantly higher levels of left ventricular ejection fraction (LVEF), stroke volume, and peak systolic velocity at the lateral mitral annulus (TDI s′-l) compared to the control group (*P* < 0.05). Conversely, the left ventricular end-systolic diameter (LVESD) and left ventricular end-systolic volume (LVESV) were significantly lower in the intensive group than in the control group (*P* < 0.05).

**Conclusion:**

In elderly patients with STEMI, high-dose rosuvastatin demonstrates superior therapeutic efficacy compared to conventional-dose therapy in alleviating inflammatory responses, improving ventricular remodeling, and enhancing cardiac function.

**Clinical Trial Registration:**

[www.chictr.org.cn], identifier [ChiCTR2200066956].

## 1 Introduction

ST-segment elevation myocardial infarction (STEMI) is the most severe form of acute coronary syndrome (ACS). It is typically caused by thrombus formation following the rupture or erosion of an atherosclerotic plaque, leading to acute and complete occlusion of a coronary artery, which results in sustained myocardial ischemia and necrosis (1). As a critical and life-threatening condition, STEMI has a sudden onset and rapid progression. Without timely and appropriate treatment, it often leads to extensive myocardial cell necrosis, which can subsequently result in ventricular remodeling and cardiac dysfunction, among other serious complications (2). Percutaneous coronary intervention (PCI) is currently a key therapeutic approach for STEMI, capable of rapidly reopening the infarct-related artery, restoring blood perfusion, and salvaging jeopardized myocardium. Although the implementation of chest pain centers has significantly shortened the door-to-wire (D-to-W) and symptom-onset to first medical contact (S-to-FMC) times for STEMI patients undergoing PCI, thereby effectively reducing mortality and disability rates, a considerable number of patients still experience ventricular remodeling and develop heart failure after PCI, particularly among elderly individuals (3). With population aging and lifestyle changes, the proportion of elderly patients with STEMI has been gradually increasing (4). This subset of patients is more susceptible to ventricular remodeling due to multiple comorbidities, poor vascular conditions, and reduced myocardial reserve, resulting in generally poorer prognoses (5). Previous studies have shown that intensive rosuvastatin therapy can significantly improve clinical outcomes in patients with coronary artery disease without increasing adverse events (6–8). However, robust evidence is still lacking regarding whether intensive statin therapy can improve ventricular remodeling and cardiac function in elderly patients undergoing emergency PCI.

The pathophysiological process of left ventricular remodeling after myocardial infarction is complex. Previous studies have demonstrated that systemic inflammation, as well as extracellular matrix remodeling and fibrosis, play critical roles in the initiation and progression of left ventricular remodeling (9). C-reactive protein (CRP), interleukin-6 (IL-6), tumor necrosis factor-α (TNF-α), and intercellular adhesion molecule-1 (ICAM-1) reflect the level of systemic inflammation (10), while Galectin-3, tissue inhibitor of metalloproteinase-4 (TIMP-4), and matrix metalloproteinase-9 (MMP-9) are associated with extracellular matrix remodeling and fibrosis (11). Therefore, the combined assessment of inflammatory markers and fibrosis-related factors may provide a more comprehensive evaluation of ventricular remodeling and the progression of heart failure following myocardial infarction.

As a guideline-recommended routine medication after STEMI, statins competitively inhibit endogenous enzymes involved in total cholesterol (TC) synthesis, thereby reducing serum low-density lipoprotein cholesterol (LDL-C) levels (12). Studies have shown that statins may exert multiple beneficial effects beyond lipid-lowering, including anti-inflammatory and antioxidant actions, improvement of endothelial function, plaque stabilization, inhibition of myocardial fibrosis, and promotion of angiogenesis (13, 14). These pleiotropic effects are thought to be mediated through mechanisms such as inhibition of isoprenylation of small G-proteins, enhancement of nitric oxide production, and modulation of various signaling pathways (15). Such pleiotropic effects may contribute to reducing ischemia-reperfusion injury, enhancing microvascular perfusion, and inhibiting ventricular remodeling in patients with STEMI. Rosuvastatin, a hydrophilic statin widely used in clinical practice, has been shown to significantly reduce the incidence and mortality of cardiovascular diseases (16). Compared to lipophilic statins such as simvastatin and atorvastatin, rosuvastatin has higher bioavailability, a lower incidence of muscle-related adverse events, and a reduced risk of drug–drug interactions (17). However, direct comparative studies between intensive and conventional statin therapy regarding their effects on ventricular remodeling and left ventricular function in elderly patients undergoing emergency PCI for STEMI remain scarce. Therefore, the primary aim of this study is to investigate the effects of intensive rosuvastatin therapy on lipid profiles, myocardial fibrosis, serum inflammatory markers, and left ventricular function in elderly patients with STEMI, in order to provide a clinical basis for optimizing treatment strategies.

## 2 Materials and methods

### 2.1 Study design

This study was a randomized, parallel-controlled, open-label trial conducted at Qingpu Branch of Zhongshan Hospital affiliated to Fudan University. The final outcome assessment was performed in a blinded manner. Laboratory testing personnel, echocardiography evaluators, and data analysts were all blinded to the treatment allocation of the patients. The study protocol was approved by the Ethics Committee of the Qingpu Branch of Zhongshan Hospital. Written informed consent was obtained from all participants prior to enrollment. All procedures were conducted in accordance with the principles outlined in the Declaration of Helsinki by the World Medical Association. The study was registered on the Chinese Clinical Trial Registry (www.chictr.org.cn) under the registration number ChiCTR2200066956.

### 2.2 Patients and drug intervention

**Figure 1:**
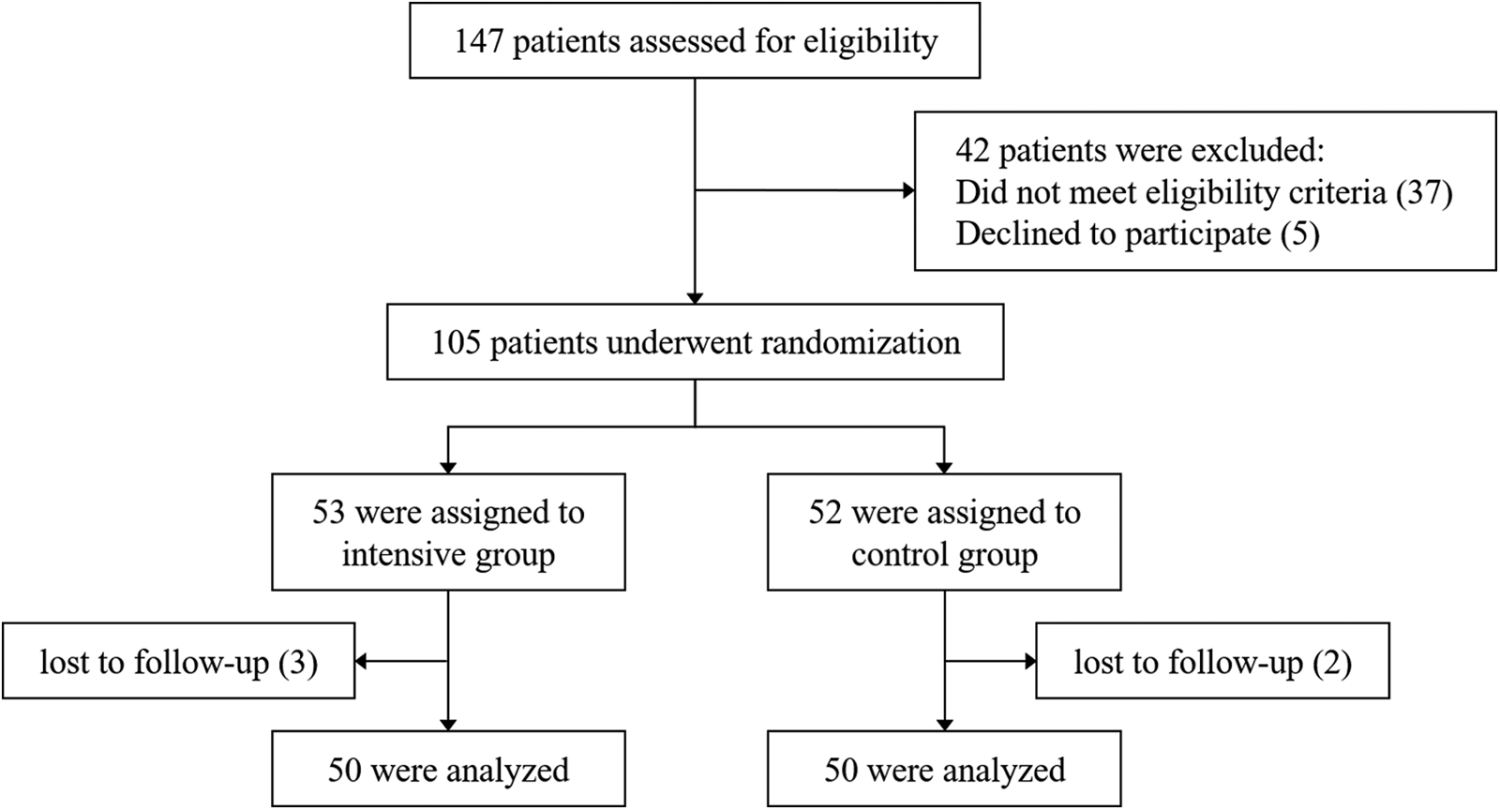
Patient enrollment and follow-up.

From March 2023 to March 2024, a total of 147 patients admitted to our hospital for acute STEMI were assessed, of whom 105 met the inclusion and exclusion criteria. Using a computer-generated randomization system, patients were randomly assigned in a 1:1 ratio to either the intensive group (rosuvastatin, 20 mg/d) or the control group (rosuvastatin, 10 mg/d). The first dose of rosuvastatin was administered prior to emergency PCI. Both groups received treatment for 8 weeks. In the end, 100 patients completed the follow-up. A detailed flowchart is shown in Figure 1. The diagnosis of STEMI was confirmed according to the 2018 European Society of Cardiology (ESC) guidelines (18). In accordance with the 2023 ESC guidelines for the management of ACS (19), all patients received guideline-directed medical therapy in addition to statin treatment, including dual antiplatelet therapy (aspirin combined with either ticagrelor or clopidogrel), β-blockers, and renin–angiotensin system inhibitors, such as angiotensin-converting enzyme inhibitors (ACEI), angiotensin II receptor blockers (ARB), or angiotensin receptor–neprilysin inhibitors (ARNI). To ensure patient compliance, monitor adverse events, and record cardiovascular events, researchers conducted telephone follow-ups every two weeks. At week 8, patients were scheduled for an outpatient visit. Medication adherence was assessed using the pill count method.

### 2.3 Inclusion and exclusion criteria

Inclusion criteria were as follows: age ≥60 years; electrocardiographic ST-segment elevation meeting the diagnostic criteria for STEMI; first occurrence of myocardial infarction; successful emergency PCI performed within 12 h of symptom onset. Exclusion criteria were as follows: severe heart failure (Killip class IV); hepatic dysfunction [alanine aminotransferase (ALT) levels persistently elevated to more than three times the upper limit of normal]; renal dysfunction [estimated glomerular filtration rate (eGFR) <30 ml/min/1.73 m^2^]; use of antioxidant agents (including coenzyme Q10 and trimetazidine) or lipid-lowering drugs (including statins, fibrates, PCSK9 inhibitors, ezetimibe, niacin and its derivatives) within 2 weeks prior to hospitalization; familial hypercholesterolemia; autoimmune diseases; malignant tumors; acute infections; severe hematologic disorders; cognitive impairment; allergy to rosuvastatin.

### 2.4 Blood index testing

Venous blood samples were collected from all patients at hospital admission and after 8 weeks of rosuvastatin treatment. Lipid profiles were assessed, including triglycerides (TG), TC, LDL-C, high-density lipoprotein cholesterol (HDL-C), apolipoprotein AI (ApoAI), apolipoprotein B (ApoB), and lipoprotein(a) [Lp(a)]. Liver function was evaluated by measuring ALT and aspartate aminotransferase (AST) levels. Additional laboratory parameters included creatine kinase (CK), high-sensitivity CRP (hs-CRP), IL-6, TNF-α, N-terminal pro–B-type natriuretic peptide (NT-proBNP), and high-sensitivity cardiac troponin T (hs-cTnT). The levels of ICAM-1, galectin-3, TIMP-4, and MMP-9 were measured using enzyme-linked immunosorbent assay (ELISA) kits (MEIMIAN, Jiangsu, China).

### 2.5 Echocardiographic assessment

Transthoracic echocardiography was performed at baseline and after 8 weeks of treatment by an experienced sonographer who was blinded to both treatment allocation and the timing of the assessments (baseline and post-treatment). For each parameter, the average value of three consecutive cardiac cycles was recorded. Left ventricular end-diastolic volume (LVEDV), left ventricular end-systolic volume (LVESV), and left ventricular ejection fraction (LVEF) were measured using the Simpson's biplane method. M-mode echocardiography in the parasternal long-axis view was used to assess left ventricular end-diastolic diameter (LVEDD), left ventricular end-systolic diameter (LVESD), and interventricular septal thickness (IVST). In the apical four-chamber view, tissue Doppler imaging (TDI) was employed to measure the peak systolic velocity at the septal (s′-s) and lateral (s′-l) mitral annulus, and the average of these two values (s′-av) was calculated.

### 2.6 Study endpoints

The primary endpoints of this study were the changes in inflammatory markers, ventricular remodeling indicators, and LVEF after 8 weeks of treatment.

### 2.7 Sample size calculation

Based on relevant literature and previous studies (20), the expected difference in mean LVEF between the two groups was set at 3.5%, with a standard deviation of 6%. Using a two-sided test with a significance level (α) of 0.05 and a power (1 − β) of 0.80, the required sample size was calculated to be 45 patients per group. Considering an approximate dropout rate of 10%, a minimum of 50 patients was required in each group.

### 2.8 Statistical analysis

Data were analyzed using IBM SPSS Statistics version 26. Variables with a normal distribution were expressed as mean ± standard deviation (SD). Between-group comparisons were performed using the independent samples *t*-test, and within-group comparisons before and after treatment were analyzed using the paired *t*-test. Non-normally distributed data were expressed as median with interquartile ranges (Q1, Q3), with between-group comparisons conducted using the Mann–Whitney *U* test and within-group comparisons using the Wilcoxon signed-rank test. Categorical variables were presented as counts and percentages. Comparisons between the two groups were performed using the chi-square test or Fisher's exact test. A *P*-value of <0.05 was considered statistically significant for all analyses.

## 3 Results

### 3.1 Baseline characteristics of the study population

A total of 147 STEMI patients aged ≥60 years were initially screened. Forty-two patients either did not meet the inclusion criteria or declined to participate. The remaining 105 patients were randomly assigned to the intensive group (*n* = 53) and the control group (*n* = 52). During the follow-up period, 3 patients in the intensive group and 2 patients in the control group were lost to follow-up. Ultimately, 50 patients in each group completed the entire study protocol and were included in the final analysis. The baseline and clinical characteristics of the participants are summarized in Table 1. The control group included 38 male and 12 female patients, with a median age of 69 [65, 77] years. The intensive group included 39 male and 11 female patients, with a median age of 69.5 [65, 76.3] years. There were no statistically significant differences between the two groups in terms of age, sex, smoking history, heart rate, blood pressure at admission, comorbidities, laboratory biochemical tests, cardiac injury and necrosis markers, or pre-admission medication use (*P* > 0.05). Additionally, no significant differences were observed in S-to-FMC time, D-to-W time, or SYNTAX scores between the two groups (*P* > 0.05). As shown in Table 2, the length of hospital stay and discharge medication regimens also did not differ significantly between the groups (*P* > 0.05).

**Table 1:** Baseline characteristics in two groups at admission.

Characteristics	Control group (*n* = 50)	Intensive group (*n* = 50)	*P* value
Age, years	69 [65, 77]	69.5 [65, 76.3]	0.945
Male, *n* (%)	38 (76%)	39 (78%)	0.812
Smoking, *n* (%)	10 (20%)	13 (26%)	0.476
Heart rate, beats/min	78.2 ± 20.7	73.5 ± 15.9	0.21
SBP, mmHg	137.1 ± 27.5	143.5 ± 27.5	0.243
DBP, mmHg	80.7 ± 15.5	83.9 ± 17.7	0.342
SYNTAX score	16.5 ± 6.6	14.9 ± 5.6	0.247
S-to-FMC time, min	134 [70.8, 292.5]	159 [112.8, 362.8]	0.154
D-to-W time, min	49.6 ± 21.5	49.2 ± 18.8	0.921
CKD stages, *n* (%)			0.441
Stage 1	21 (42%)	28 (56%)	
Stage 2	24 (48%)	20 (40%)	
Stage 3a	3 (6%)	1 (2%)	
Stage 3b	2 (4%)	1 (2%)	
Infarct related artery, *n* (%)			0.678
Left anterior descending	25 (50%)	21 (42%)	
Left circumflex	5 (10%)	7 (14%)	
Right coronary artery	20 (40%)	22 (44%)	
Killip class, *n* (%)			0.749
I	34 (68%)	31 (62%)	
II	12 (24%)	13 (26%)	
III	4 (8%)	6 (12%)	
Medical history, *n* (%)			
Hypertension	33 (66%)	25 (50%)	0.105
Diabetes mellitus	11 (22%)	9 (18%)	0.617
Hyperlipemia	8 (16%)	6 (12%)	0.564
Atrial fibrillation	2 (4%)	3 (6%)	0.646
Hematological and biochemical variables			
WBC, 10^9^/L	8.5 ± 3.1	7.7 ± 2.1	0.166
RBC, 10^12^/L	4.4 ± 0.6	4.3 ± 0.5	0.42
Platelet, 10^9^/L	221.5 ± 77.5	205.7 ± 51.3	0.234
Hemoglobin, g/L	133.5 ± 18.2	130.3 ± 12.9	0.304
ALT, U/L	34 [25, 49.5]	31 [21, 42]	0.147
AST, U/L	57.5 [32.5, 160.5]	56.5 [34.5, 119.5]	0.783
Serum creatinine, umol/L	72.9 ± 18	75.7 ± 36	0.741
Serum uric acid, umol/L	316.9 ± 84.7	308.5 ± 90	0.633
Blood urea nitrogen, mmol/L	5.8 ± 1.7	5.8 ± 1.9	0.956
eGFR, ml/min/1.73 m^2^	86.3 ± 20.9	87.6 ± 15.8	0.725
FBG, mmol/L	7.5 [6.3, 11.3]	6.9 [5.7, 8.7]	0.082
HbA1c, %	6.2 [5.6, 8.6]	6.1 [5.7, 7.2]	0.828
D-dimer, mg/L	0.67 [0.38, 1.6]	0.61 [0.32, 1.21]	0.334
Cardiac injury biomarkers			
CK-MB, ng/ml	7.2 [2.7, 40.9]	4.2 [2.2, 20.6]	0.163
Myoglobin, ng/ml	66.5 [31.3, 226.5]	55.5 [30.5, 175.5]	0.785
hs-cTnT, ng/L	20 [10, 107.8]	18.5 [10, 91]	0.382
NT-proBNP, pg/ml	298 [87.8, 2,464.5]	332 [99.3, 1,314.3]	0.629
Peak hs-cTnT, ng/L	2,564 [319.3, 7,452.3]	1,985 [321.8, 5,555.3]	0.491
Peak NT-proBNP, pg/ml	1,042.5 [290.3, 3,447.8]	1,224.5 [482.8, 2,718.8]	0.756
Pre-admission medications, *n* (%)			
ACEI/ARB/ARNI	11 (22%)	7 (14%)	0.298
β-blocker	4 (8%)	2 (4%)	0.4
SGLT2i	4 (8%)	3 (6%)	0.695
GLP-1RA	2 (4%)	2 (4%)	1
NOAC	2 (4%)	3 (6%)	0.646

SBP, systolic pressure; DBP, diastolic pressure; CKD, chronic kidney disease; S-to-FMC, symptom onset to first medical contact; D-to-W, door to wire; WBC, white blood cell; RBC, red blood cell; ALT, alanine transaminase; AST, aspartate transaminase; eGFR, estimated glomerular filtration rate; FBG, fasting blood glucose; HbA1c, glycated hemoglobin; CK-MB, creatine kinase myocardial band; hs-cTnT, high-sensitivity cardiac troponin T; NT-proBNP, N-terminal brain natriuretic peptide precursor; ACEI, angiotensin-converting enzyme inhibitor; ARB, angiotensin II receptor blocker; ARNI, angiotensin receptor-neprilysin inhibitor; SGLT2i, sodium-glucose cotransporter 2 inhibitor; GLP-1RA, glucagon-like peptide-1 receptor agonist; NOAC, novel oral anticoagulants.

**Table 2:** Comparison of length of hospital stay and discharge medication between the two groups.

Characteristics	Control group (*n* = 50)	Intensive group (*n* = 50)	*P* value
Length of hospital stay, days	7 [7, 8]	7 [6, 8]	0.257
Aspirin, *n* (%)	50 (100%)	50 (100%)	1
Ticagrelor, *n* (%)	28 (56%)	31 (62%)	0.542
Clopidogrel, *n* (%)	22 (44%)	19 (38%)	0.542
ACEI/ARB/ARNI, *n* (%)	47 (94%)	48 (96%)	0.646
β-blocker, *n* (%)	42 (84%)	45 (90%)	0.372
MRA, *n* (%)	6 (12%)	9 (18%)	0.401
SGLT2i, *n* (%)	15 (30%)	19 (38%)	0.398
GLP-1RA, *n* (%)	6 (12%)	8 (16%)	0.564
NOAC, *n* (%)	4 (8%)	3 (6%)	0.695

ACEI, angiotensin-converting enzyme inhibitor; ARB, angiotensin II receptor blocker; ARNI, angiotensin receptor-neprilysin inhibitor; MRA, mineralocorticoid receptor antagonist; SGLT2i, sodium-glucose cotransporter 2 inhibitor; GLP-1RA, glucagon-like peptide-1 receptor agonist; NOAC, novel oral anticoagulants.

### 3.2 Changes in lipid profiles, liver function, and CK levels

Before treatment, there were no statistically significant differences between the two groups in lipid parameters, including TG, TC, LDL-C, HDL-C, ApoAI, ApoB, and Lp(a) (*P* > 0.05). After 8 weeks of rosuvastatin therapy, both groups showed significant reductions in TG, TC, LDL-C, ApoB, and Lp(a) levels (*P* < 0.05), as well as significant increases in HDL-C levels (*P* < 0.05), while ApoAI levels remained unchanged (*P* > 0.05). After treatment, the levels of TG, TC, and LDL-C were lower in the intensive group compared to the control group, but the differences were not statistically significant (*P* > 0.05). There were no significant differences in HDL-C, ApoAI, ApoB, and Lp(a) levels between the two groups after treatment (Table 3).

**Table 3:** Comparison of lipid profiles and liver enzymes at baseline and after treatment.

Parameters		Control group (*n* = 50)	Intensive group (*n* = 50)	*P*-value
TG, mmol/L	Baseline	1.58 ± 0.69	1.63 ± 0.56	0.702
	After 8 weeks	1.22 ± 0.52	1.15 ± 0.4	0.427
	*P*-value	<0.001	<0.001	
TC, mmol/L	Baseline	5.93 ± 0.69	6 ± 0.68	0.601
	After 8 weeks	4.24 ± 0.56	4.09 ± 0.5	0.146
	*P*-value	<0.001	<0.001	
LDL-C, mmol/L	Baseline	3.31 ± 0.59	3.2 ± 0.61	0.374
	After 8 weeks	2.01 ± 0.4	1.86 ± 0.37	0.059
	*P*-value	<0.001	<0.001	
HDL-C, mmol/L	Baseline	1.13 ± 0.32	1.11 ± 0.28	0.732
	After 8 weeks	1.21 ± 0.28	1.23 ± 0.3	0.707
	*P*-value	0.012	<0.001	
ApoAI, g/L	Baseline	1.31 ± 0.3	1.29 ± 0.34	0.757
	After 8 weeks	1.34 ± 0.3	1.33 ± 0.27	0.502
	*P*-value	0.153	0.074	
ApoB, g/L	Baseline	0.97 ± 0.25	0.93 ± 0.22	0.422
	After 8 weeks	0.78 ± 0.22	0.71 ± 0.2	0.075
	*P*-value	<0.001	<0.001	
Lp(a), mg/L	Baseline	300 [140.3, 450]	319 [131.3, 667.3]	0.551
	After 8 weeks	214.5 [100.3, 315]	183.5 [102.3, 292.5]	0.444
	*P*-value	<0.001	<0.001	
ALT, U/L	Baseline	34 [24.8, 50]	31 [21, 42]	0.147
	After 8 weeks	32.5 [27, 49.3]	29 [22, 42.8]	0.212
	*P*-value	0.158	0.628	
AST, U/L	Baseline	57.5 [32.5, 160.5]	56.5 [34.5, 119.5]	0.783
	After 8 weeks	38 [24.3, 49.8]	34 [29, 44.5]	0.92
	*P*-value	<0.001	<0.001	
CK, U/L	Baseline	165 [97.3, 284.5]	172 [102.8, 291.8]	0.73
	After 8 weeks	84.5 [68.8, 118.3]	98 [79.5, 143.5]	0.074
	*P*-value	<0.001	<0.001	

TG, triglyceride; TC, total cholesterol; LDL-C, low-density lipoprotein cholesterol; HDL-C, high-density lipoprotein cholesterol; ApoAI, apolipoprotein AI; ApoB, apolipoprotein B; Lp(a), lipoprotein(a); ALT, alanine transaminase; AST, aspartate transaminase; CK, creatine kinase.

Before treatment, no statistically significant differences were observed between the two groups in liver function markers (ALT, AST) or CK levels (*P* > 0.05). Compared with baseline, both groups exhibited significant decreases in AST and CK levels after treatment (*P* < 0.05), while ALT levels showed no significant change (*P* > 0.05). After treatment, there were no significant differences in ALT and AST levels between the intensive and control groups (*P* > 0.05). Although CK levels were higher in the intensive group than in the control group, the difference was not statistically significant (*P* > 0.05) (Table 3). In addition, two patients in the intensive group and one patient in the control group experienced myalgia after treatment, with no significant difference between the groups (*P* > 0.05). No major adverse cardiovascular events (MACE), such as repeat PCI, hospitalization for heart failure, or cardiac death, were observed in either group.

### 3.3 Changes in serum inflammatory factors

Before treatment, there were no significant differences in the concentrations of hs-CRP, IL-6, TNF-α, and ICAM-1 between the two groups (*P* > 0.05). After treatment, serum levels of hs-CRP, IL-6, TNF-α, and ICAM-1 decreased in both groups (*P* < 0.05), with the reductions being more pronounced in the intensive group compared to the control group (*P* < 0.05) (Table 4).

**Table 4:** Comparison of inflammatory factor levels at baseline and after treatment.

Parameters		Control group (*n* = 50)	Intensive group (*n* = 50)	*P*-value
hs-CRP, mg/L	Baseline	7.4 [3.7, 12.6]	6.6 [3.1, 11.8]	0.662
	After 8 weeks	3.9 [2.8, 6.7]	3 [1.8, 4.7]	0.028
	*P*-value	<0.001	<0.001	
IL-6, pg/ml	Baseline	4.7 [3, 7.6]	4 [2.1, 6.3]	0.187
	After 8 weeks	3.1 [2, 4.1]	2.3 [1.5, 3.4]	0.035
	*P*-value	<0.001	<0.001	
TNF-α, pg/ml	Baseline	7.4 [5.7, 9.8]	7.3 [5.6, 8.8]	0.815
	After 8 weeks	5 [4.2, 5.7]	4.3 [3.3, 5]	0.01
	*P*-value	<0.001	<0.001	
ICAM-1, ng/ml	Baseline	554.2 ± 98.3	549.1 ± 90.8	0.788
	After 8 weeks	367.7 ± 66.6	319.2 ± 69.6	0.001
	*P*-value	<0.001	<0.001	

hs-CRP, high-sensitivity C-reactive protein; IL-6, interleukin-6; TNF-α, tumor necrosis factor-α; ICAM-1, intercellular cell adhesion molecule-1.

### 3.4 Changes in indicators of ventricular remodeling and myocardial fibrosis

Before treatment, there were no significant differences in NT-proBNP, hs-cTnT, galectin-3, TIMP-4, and MMP-9 levels between the two groups (*P* > 0.05). After treatment, levels of NT-proBNP, hs-cTnT, Galectin-3, and MMP-9 in both groups significantly decreased (*P* < 0.05), while TIMP-4 levels significantly increased (*P* < 0.05). Post-treatment comparisons between groups showed that the intensive group had significantly lower levels of NT-proBNP, Galectin-3, and MMP-9 compared to the control group (*P* < 0.05), and significantly higher TIMP-4 levels (*P* < 0.05). There was no statistically significant difference in hs-cTnT levels between the two groups (*P* > 0.05) (Table 5).

**Table 5:** Comparison of ventricular remodeling indicators before and after treatment.

Parameters		Control group (*n* = 50)	Intensive group (*n* = 50)	*P*-value
NT-proBNP, pg/ml	Baseline	1,042.5 [290.3, 3,447.8]	1,224.5 [482.8, 2,718.8]	0.723
	After 8 weeks	308 [95.8, 625]	100 [61, 283.3]	0.003
	*P*-value	<0.001	<0.001	
hs-cTnT, ng/L	Baseline	2,564 [319.3, 7,452.3]	1,985 [321.8, 5,555.3]	0.491
	After 8 weeks	10.1 [8, 14]	9.2 [7, 11.4]	0.18
	*P*-value	<0.001	<0.001	
Galectin-3, ng/ml	Baseline	21.4 ± 3.1	20.6 ± 3.7	0.253
	After 8 weeks	14.8 ± 2.2	11.8 ± 2.6	<0.001
	*P*-value	<0.001	<0.001	
TIMP-4, ng/ml	Baseline	41.1 ± 4.2	42.5 ± 4.1	0.108
	After 8 weeks	66.2 ± 9.1	71.8 ± 8.3	0.002
	*P*-value	<0.001	<0.001	
MMP-9, ng/ml	Baseline	284.9 ± 65.9	291.7 ± 62	0.597
	After 8 weeks	176.5 ± 40.4	145.3 ± 34.1	<0.001
	*P*-value	<0.001	<0.001	

NT-proBNP, N-terminal brain natriuretic peptide precursor; hs-cTnT, high-sensitivity cardiac troponin T; TIMP-4, tissue inhibitor of metalloproteinase-4; MMP-9, matrix metalloproteinase-9.

### 3.5 Changes in echocardiographic parameters

At baseline, there were no significant differences in all measured parameters between the two groups (*P* > 0.05). After 8 weeks of treatment, both groups showed a downward trend in LVESD, LVEDD, LVESV, and LVEDV compared to baseline (*P* < 0.05), while LVEF, stroke volume, and TDI parameters (TDI s′-s, TDI s′-l, and TDI s′-av) improved (*P* < 0.05). Intergroup comparisons after treatment revealed that the intensive group had significantly lower LVESD and LVESV values (*P* < 0.05), and significantly higher LVEF and stroke volume compared to the control group (*P* < 0.05). Furthermore, improvement in TDI s′-l was greater in the intensive group than in the control group (*P* < 0.05). Although heart rate decreased significantly from baseline in both groups (*P* < 0.05), there was no significant difference between the two groups after treatment (*P* > 0.05). After 8 weeks, no significant differences were observed between the two groups in cardiac output or IVST (*P* > 0.05) (Table 6).

**Table 6:** Comparison of echocardiographic measurements at baseline and after treatment.

Parameters		Control group (*n* = 50)	Intensive group (*n* = 50)	*P*-value
LVESD, mm	Baseline	34.1 ± 5.2	35 ± 5	0.388
	After 8 weeks	31.1 ± 5.1	29 ± 3.7	0.024
	*P*-value	<0.001	<0.001	
LVEDD, mm	Baseline	51.3 ± 4.7	51.8 ± 4.9	0.602
	After 8 weeks	49.3 ± 4.7	48 ± 4.4	0.151
	*P*-value	<0.001	<0.001	
LVESV, ml	Baseline	52.1 ± 12.2	52.9 ± 12.7	0.748
	After 8 weeks	46 ± 11.4	41.3 ± 9.4	0.029
	*P*-value	<0.001	<0.001	
LVEDV, ml	Baseline	109.6 ± 14.7	108.6 ± 13.9	0.722
	After 8 weeks	106.8 ± 14.1	106.2 ± 12.6	0.822
	*P*-value	<0.001	<0.001	
LVEF, %	Baseline	52.6 ± 7.9	51.4 ± 8.3	0.467
	After 8 weeks	57.3 ± 7.4	61.5 ± 6.2	0.003
	*P*-value	<0.001	<0.001	
Stroke volume, ml	Baseline	57.5 ± 10.4	55.7 ± 9.9	0.373
	After 8 weeks	60.9 ± 9.9	65.8 ± 10.3	0.017
	*P*-value	<0.001	<0.001	
Heart rate, beats/min	Baseline	74.9 ± 12.7	77.5 ± 10.3	0.276
	After 8 weeks	68.4 ± 8.1	67.5 ± 8.1	0.556
	*P*-value	<0.001	<0.001	
Cardiac output, ml/min	Baseline	4,270.5 ± 887.8	4,297.3 ± 889.1	0.88
	After 8 weeks	4,186.4 ± 698.7	4,444 ± 905	0.114
	*P*-value	0.288	0.129	
IVST, mm	Baseline	10.2 ± 0.6	10.3 ± 0.7	0.883
	After 8 weeks	10.1 ± 0.7	10.1 ± 0.8	0.891
	*P*-value	0.109	0.107	
TDI s'-s, cm/s	Baseline	6.6 ± 1.8	6.6 ± 1.5	0.88
	After 8 weeks	7.1 ± 1.6	7.4 ± 1.4	0.24
	*P*-value	<0.001	<0.001	
TDI s'-l, cm/s	Baseline	8 ± 1.7	8.1 ± 1.8	0.752
	After 8 weeks	8.7 ± 1.6	9.3 ± 1.4	0.044
	*P*-value	<0.001	<0.001	
TDI s'-av, cm/s	Baseline	7.3 ± 1.7	7.4 ± 1.5	0.826
	After 8 weeks	7.9 ± 1.5	8.4 ± 1.2	0.081
	*P*-value	<0.001	<0.001	

LVESD, left ventricular end-systolic diameter; LVEDD, left ventricular end-diastolic diameter; LVESV, left ventricular end-systolic volume; LVEDV, left ventricular end-diastolic volume; LVEF, left ventricular ejection fraction; IVST, interventricular septum thickness; TDI s′-s, tissue Doppler imaging s′-septal; TDI s′-l, tissue Doppler imaging s′-lateral; TDI s′-av, tissue Doppler imaging s′-average.

## 4 Discussion

Patients with STEMI are prone to ventricular remodeling and heart failure due to acute myocardial ischemic injury. Studies have shown that rosuvastatin may improve left ventricular remodeling through its anti-inflammatory and antifibrotic effects (20). According to the recommendations of the American College of Cardiology (ACC) and the ESC, high-intensity rosuvastatin (20 mg/d) offers superior lipid-lowering efficacy and cardiovascular protection for STEMI patients (21, 22). The results of this study indicate that, compared with moderate-intensity rosuvastatin (10 mg/d), a dosage of 20 mg/d is more effective in attenuating the postoperative inflammatory response, suppressing ventricular remodeling, and improving cardiac function in elderly patients undergoing PCI, without causing significant adverse effects on liver function.

Patients with acute myocardial infarction often exhibit lipid metabolism disorders, characterized by elevated levels of LDL-C, TG, TC, and Lp(a), along with reduced HDL-C levels (23). LDL-C is an independent risk factor for acute myocardial infarction, and patients with elevated LDL-C levels have a higher risk of recurrent myocardial infarction during long-term follow-up (24, 25). ApoB is the core protein of atherogenic lipoproteins such as LDL. In this study, after 8 weeks of rosuvastatin treatment, both groups showed significant improvements in lipid profiles. Although the differences between the two groups were not statistically significant, the reductions in LDL-C and ApoB were slightly greater in the intensive group, suggesting a potential advantage for secondary prevention of cardiovascular events. A previous study on elderly Chinese patients with coronary heart disease compared the effects of 10 mg and 20 mg rosuvastatin. After four months of treatment, there were no significant differences in lipid parameters (including LDL-C, TG, TC, and HDL-C) between the two groups, and the incidence of adverse events was similar (26). These findings are consistent with our results. It should be noted that acute myocardial infarction can lead to significant elevations in AST and CK levels. Our study demonstrated that high-dose rosuvastatin had no adverse effect on liver function and did not lead to increased ALT or AST levels. There was also no significant difference in CK levels between the two groups. These findings are supported by a study conducted by Taherkhani et al., in which 110 elderly patients received 40 mg/d rosuvastatin for 6 weeks. The safety assessment showed that only two patients experienced myalgia, 12 had muscle cramps, no cases of jaundice were observed, and no patients discontinued treatment due to adverse events (6). Therefore, high-dose rosuvastatin appears to be well tolerated in elderly patients with STEMI.

Inflammatory factors are closely associated with coronary artery disease, and changes in serum inflammatory markers can help predict the prognosis of patients with cardiovascular disease. TNF-α and IL-6 are key pro-inflammatory cytokines involved in the pathophysiological response following myocardial infarction. Studies have shown that their levels increase significantly after myocardial infarction and contribute to the expansion of myocardial injury through direct cytotoxic effects or by inducing inflammatory responses (27). Hs-CRP is an acute-phase protein synthesized by the liver in response to IL-6 stimulation. It participates in the pathological process following myocardial infarction by promoting inflammatory cell infiltration and affecting endothelial function (28). Elevated hs-CRP levels after myocardial infarction are associated with left ventricular remodeling, the development of heart failure, and an increased risk of mortality (29, 30). A meta-analysis involving 18,715 patients with acute myocardial infarction who underwent PCI demonstrated that elevated CRP levels were associated with an increased risk of in-hospital and short-term all-cause mortality, as well as significantly higher rates of cardiovascular mortality and MACE (31). The potential mechanisms may include CRP-mediated inflammatory responses that exacerbate plaque instability, microvascular dysfunction, and myocardial remodeling. One study evaluated the effect of a single preoperative 40 mg dose of rosuvastatin on the acute inflammatory response after PCI in patients with stable coronary artery disease. Compared with patients who did not receive pretreatment, those receiving high-dose rosuvastatin had significantly reduced serum levels of IL-6 and hs-CRP (32). In addition, serum ICAM-1 levels are significantly elevated in patients with acute myocardial infarction (33). ICAM-1 mediates the adhesion of neutrophils to cardiomyocytes, facilitating the release of proteases, reactive oxygen species, and other cytotoxic substances that exacerbate myocardial injury and promote adverse remodeling (34). The results of this study showed that, compared with the control group, the intensive group achieved significantly greater reductions in serum hs-CRP, IL-6, TNF-α, and ICAM-1 levels, indicating a more effective suppression of the postoperative inflammatory response.

Galectin-3 is a β-galactoside-binding lectin that plays a key role in myocardial fibrosis and inflammatory processes. Researches have shown that galectin-3 can activate macrophages, promote the proliferation of cardiac fibroblasts, and stimulate collagen synthesis (35). Furthermore, elevated levels of galectin-3 are associated with the severity of heart failure and poor clinical outcomes (36). A randomized controlled trial compared the effects of high-dose atorvastatin (80 mg/d) and rosuvastatin (40 mg/d) on serum galectin-3 levels in patients with acute myocardial infarction. The results showed that patients in the rosuvastatin group experienced a significant reduction in galectin-3 levels after 4 weeks of treatment, whereas no significant change was observed in the atorvastatin group (37), suggesting that rosuvastatin may have a stronger effect in modulating biomarkers related to myocardial fibrosis. MMP-9 and TIMP-4 belong to the matrix metalloproteinases (MMPs) and tissue inhibitor of metalloproteinases (TIMPs) protein families, respectively. MMPs are primarily responsible for extracellular matrix degradation, and their increased activity has been linked to ventricular dilation and cardiac dysfunction (38). TIMPs are natural antagonists of MMPs, capable of inhibiting MMPs activity and maintaining the structural integrity of the myocardium (39). In the present study, after 8 weeks of treatment, the intensive group had significantly higher TIMP-4 levels and significantly lower levels of MMP-9 and galectin-3 compared to the control group. NT-proBNP is a key biomarker for evaluating cardiac function and ventricular remodeling after myocardial infarction. In this study, high-dose rosuvastatin produced a more pronounced reduction in NT-proBNP levels. Therefore, rosuvastatin at 20 mg/d may be more effective than 10 mg/d in reducing myocardial fibrosis and improving cardiac function.

Heart failure is one of the most common complications in patients with STEMI and has a significant impact on prognosis. Previous studies have shown that high-intensity statin therapy may improve clinical outcomes through its anti-inflammatory effects. Gavazzoni et al. compared the efficacy of high-dose vs. moderate-dose atorvastatin in patients with STEMI (40). The results indicated that high-dose treatment led to more significant reductions in inflammatory markers (such as hs-CRP and IL-6) and greater improvements in endothelial function. These findings suggest that intensive statin therapy may exert stronger anti-inflammatory and vascular protective effects in the early phase of myocardial infarction, supporting its role in delaying ventricular remodeling and preventing heart failure. In a meta-analysis conducted by Sun et al., three cohort studies and four randomized controlled trials were evaluated to compare the effects of rosuvastatin combined with ticagrelor vs. ticagrelor alone in patients undergoing PCI (41). The findings indicated that the combination of rosuvastatin and ticagrelor not only significantly reduced the incidence of MACE compared to ticagrelor monotherapy, but also improved left ventricular structure and function (including LVESD, LVEDD, and LVEF) and lowered NT-proBNP levels. These findings highlight the potential of rosuvastatin in myocardial protection and heart failure prevention. Notably, improvement in ventricular function carries clear clinical significance. Breathett et al. demonstrated that even a modest increase in LVEF can predict a lower risk of all-cause mortality and is associated with a reduced risk of hospitalization for heart failure (42). In our study, echocardiographic results showed that LVEF, TDI s′-l, and stroke volume were significantly higher in the intensive group than in the control group after treatment, indicating improved cardiac systolic function. This suggests that high-dose rosuvastatin may be more effective than conventional doses in improving ventricular remodeling and cardiac function. Given that there were no significant differences in lipid levels between the two groups after treatment, rosuvastatin may exert cardioprotective effects independent of its lipid-lowering properties.

This study has several limitations. Firstly, as a single-center study with a relatively small sample size, the statistical power and generalizability of the findings may be limited. Secondly, although efforts were made to ensure the completeness of data collection, some data were missing—for example, a few patients did not complete follow-up—which may have introduced bias in the analysis of certain parameters. In addition, the follow-up period was relatively short, and the primary endpoints focused mainly on inflammatory markers, indicators of ventricular remodeling, and cardiac function parameters, without including long-term clinical outcomes such as cardiac death, heart failure hospitalization, or MACE. Therefore, the assessment of clinical benefits requires further validation. Since this study only evaluated the differences between high-dose and standard-dose rosuvastatin, without considering a comparison with other high-intensity statins (such as atorvastatin), the comprehensiveness of the results may be limited. Future studies with multicenter designs, larger sample sizes, comparisons across different statins, and longer follow-up durations are needed to confirm these findings.

## 5 Conclusion

In elderly patients with STEMI undergoing PCI, high-dose rosuvastatin demonstrated superior efficacy compared to the conventional dose in reducing inflammation, attenuating myocardial fibrosis, and improving ventricular remodeling and cardiac function. Its cardioprotective effects may be independent of lipid-lowering mechanisms.

## Data Availability

The raw data supporting the conclusions of this article will be made available by the authors, without undue reservation.
